# Co-Cultivation Assays for Detecting Infectious Human-Tropic Porcine Endogenous Retroviruses (PERVs)

**DOI:** 10.3390/ijms26157111

**Published:** 2025-07-23

**Authors:** Joachim Denner

**Affiliations:** Institute of Virology, Free University Berlin, 14163 Berlin, Germany; joachim.denner@fu-berlin.de; Tel.: +49-175-591-7006

**Keywords:** porcine endogenous retroviruses, infection assays, xenotransplantation, regulatory bodies

## Abstract

Porcine endogenous retroviruses (PERVs) are integrated into the genome of all pigs. As they can be released as infectious virus particles capable of infecting human cells in vitro, they pose a potential risk for xenotransplantation involving pig cells or organs. To assess whether pigs produce infectious human-tropic viruses, infection assays with human cells are required. There are three main types of assays. First is the incubation of human target cells with gamma-irradiated pig cells. This method ensures that viral transmission is assessed in the absence of replicating pig cells. However, gamma irradiation may alter gene expression in pig cells, potentially affecting the results. Second is the co-culture in a double-chamber system in which pig and human cells are separated by a porous membrane, preventing direct cell-to-cell contact. While this method allows for the detection of infection by free virus particles, it does not account for infection via cell-to-cell transmission, which is a common mode of retroviral infection. And third is the co-culture of pig cells with human cells expressing a resistance gene. The resistance gene allows selective elimination of pig cells upon the addition of a selection medium. This assay enables both free virus and cell-to-cell transmission as well as complete removal of pig cells, which may not be fully achieved in the first type of assay. The third assay best simulates the conditions of in vivo xenotransplantation. However, in all cases the selection of donor and recipient cells is crucial to the experimental outcome. Results only indicate whether a specific pig cell type releases PERVs and whether a specific human cell type is susceptible to infection. A negative infection result does not necessarily reflect the in vivo situation, in which a transplanted organ consists of multiple pig cell types interacting with a diverse range of human cells within a living organism. Knowledge of these limitations is important for authorities regulating clinical applications for xenotransplantation.

## 1. Introduction

Porcine endogenous retroviruses (PERVs) are gammaretroviruses integrated in the genome of pigs; PERV-A and PERV-B are found in all pigs and PERV-C in many, but not all, pigs [1]. PERV-A and PERV-B have been found to infect human cells in culture (human-tropic viruses), especially tumor and immortal cells [2,3]. There are only a few reports on the infection of primary cells [4,5,6]. PERV-C does not infect human cells, only pig cells (*Ecotropic virus*). However, recombinations between PERV-A and PERV-C have been detected in living pigs. In these cases, PERV-C acquired the receptor binding site for human PERV receptors from PERV-A through recombination in the *env* gene. Consequently, PERV-A/C is capable of infecting human cells. Each PERV-A/C variant exhibits unique recombination sites [7]. PERV-A/C replicates at higher titers on human cells compared with the parental PERV-A [8,9,10,11]. As these viruses are integrated into the genome, they cannot be eliminated as other pig viruses can. Therefore, they pose a special risk for xenotransplantation using pig cells, tissues, or organs. To detect PERVs and analyze their expression, several methods have been developed, mainly PCRs and real-time PCRs for the detection of proviruses, reverse transcriptase PCRs for the detection of viral mRNA, immunofluorescence for the detection of viral proteins, and electron microscopy for the detection of virus particles [12,13,14]. Microarrays and next-generation sequencing (NGS)-based methods should be developed to improve screening. For example, Kono et al. [15] developed a highly sensitive method for the detection of PERV-A/C using NGS technologies. They found that NGS analysis allowed the detection of PERV-A/C at abundance ratios of 1% and 0.1% with true positive rates of 100% and 57%, respectively, indicating that it would be useful for the rapid detection of PERV-A/C emergence after the transplantation of porcine products [15].

However, the main question, regarding which pigs release infectious human-tropic viruses able to infect human cells, is difficult to answer. Neither the number of integrated proviruses (some of which are defective), nor levels of RNA or protein expression, nor the release of viral particles determines the infectivity of the virus [16]. For example, PK15 cells treated with clustered regularly interspaced short palindromic repeats/CRISPR associated (CRISPR/Cas) to specifically inactivate a highly conserved region of the polymerase gene of PERVs still produce viral particles. Although these particles may theoretically enter human cells—since they retain envelope proteins on their surface—they are non-infectious because they lack the ability to generate a DNA provirus for integration into the host genome [17]. The question of infectivity can only be resolved through infection assays, which are difficult to establish and, as is discussed below, have extremely limited utility. In the following section, three commonly used assays and their limitations are examined.

## 2. Co-Cultivation with Pig Cells After Gamma Irradiation

**Figure 1:**
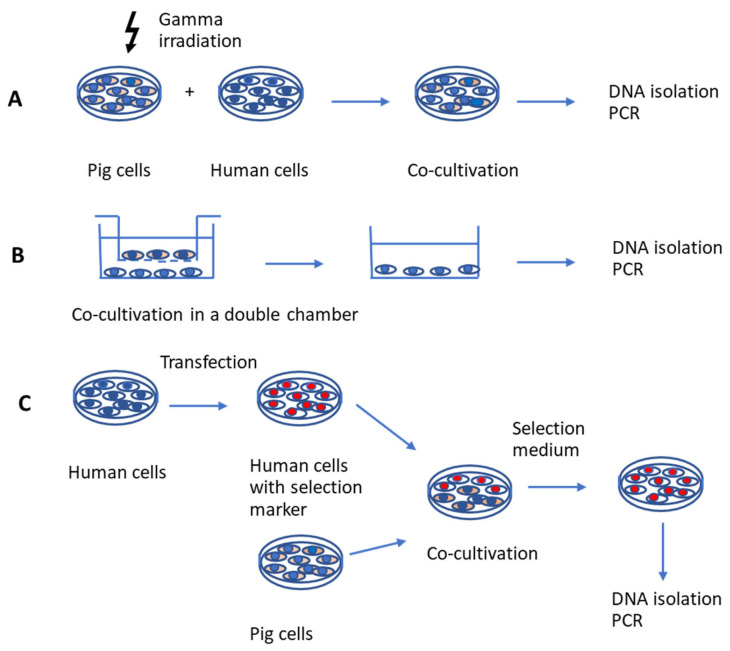
Schematic presentation of three co-cultivation strategies to study PERVs infection of human cells. (**A**) Pig cells are treated with gamma irradiation and co-cultured with human cells. (**B**) Pig cells and human cells are incubated in a double chamber system, and the chamber with pig cells is removed. (**C**) Human cells are transfected with a selection marker, co-cultured with pig cells, and selection medium is added. In all cases, DNA is isolated from human cells and a PCR is performed to detect integrated PERVs sequences. In addition to a PCR, other methods of PERVs detection could be used, e.g., detection of enzymatically active reverse transcriptase or detection of virus particles by electron microscopy.

The first type of assay involves incubating human cells with gamma-irradiated pig cells (Figure 1A). Gamma irradiation, i.e., exposure to ionizing radiation with gamma rays, inhibits the proliferation of pig cells [18,19,20] and allows the isolation and analysis of living human cells after co-cultivation. The effects of gamma irradiation on cell viability depend on the time of exposure and culture conditions. Unfortunately, it may lead to an incomplete elimination of porcine cells and consequently to false-positive infection results. When a mouse macrophage cell line was treated by gamma irradiation, the activation of NF-κB and NLRP3 signaling was induced, thereby promoting the expression of pro-inflammatory cytokines [21], indicating that some functions remained. However, gamma irradiation may have a negative effect on virus release.

This method was used when gamma irradiated (100 Gy) porcine kidney PK15 cells, which produce PERVs, were incubated with several human cell lines. Using this method it was demonstrated for the first time that PERVs could infect human cells, highlighting a potential risk for xenotransplantation involving pig cells, tissues, or organs [2]. In addition to human embryonic kidney 293 cells, MRC-5 fetal diploid fibroblasts, the rhabdomyosarcoma cell line RD, a B-cell line, and two T-lymphocytic cell lines became productively infected. This method was also used when, for the first time, a human-tropic PERV released from pig lymphocytes irradiated with 2000 rads from a 137Cs source was shown to infect human cells [8]. This virus was later identified as a recombinant PERV-A/C [9]. Furthermore, Garkavenko et al. [22] co-cultivated gamma irradiated (2000 rads from a 137Cs source) pig peripheral blood mononuclear cells (PBMCs) or PK15 cells with human 293 cells. The PBMCs were from a designated pathogen-free (DPF) herd of New Zealand pigs and Auckland Island pigs, and among them were PERV-C-positive pigs. No transmission of human-tropic PERVs was observed in contrast to PK15 cells.

## 3. Effects of Gamma Irradiation on Donor Pig Cells

Ionizing radiation induces cellular damage, as indicated by DNA damage, and reductions in DNA damage repair and cell survival. Other indicators of cellular damage may also include cell cycle alterations, glycolysis effects, and cell apoptosis [18]. The DNA damage induced by irradiation could result in direct and indirect biological effects associated with reactive oxygen species (ROS). Increased ROS, DNA double strand breaks (DSBs), cellular apoptosis, and inhibition of cell proliferation were observed [18,19]. Gamma radiation at a dose as low as 5 Gy can cause DNA strand breaks and chromosome aberrations. Irradiated PBMCs retain cellular functions, such as cytokine secretion, cytotoxicity, and co-stimulation, but are unable to proliferate [20]. Irradiated PBMCs and pig embryonic kidney cells also retain the ability to release PERVs [8,22]. Due to the negative effect on the DNA and subsequently on gene and virus expression, as well as the potential impossibility to eliminate all pig cells, this scenario is significantly different from the situation in xenotransplantation.

## 4. Separation of Pig and Human Cells by Porous Membranes

The second assay used to study PERVs release and infection of human cells is based on co-cultivation of pig and human cells using a double-chamber system (Figure 1B). Human cells are located in the lower chamber and pig cells in the upper one, separated by a porous membrane. This membrane, with a pore size of 0.4 µm, allows the exchange of medium, small molecules, and viruses. In this scenario all cells are alive during the whole co-cultivation. This method was employed to investigate the infectivity of PERVs expressed by PBMCs purified from Göttingen minipigs and activated with the T cell mitogen phytohemagglutinin (PHA). PBMCs were co-cultivated with permissive human 293 cells to assess trans-species transmission potential [23]. Except in positive control experiments—where 293 cells producing high-titer PERV-A/C were co-cultivated with naïve 293 cells—no proviral DNA was detected in the target 293 cells. Although PHA stimulation increased PERVs expression in porcine PBMCs, no human-tropic virus was released [23].

This method was also applied in subsequent experiments in which PHA-activated human PBMCs, 293 cells, and pig ST-Iowa cells were seeded in 6-well plates, and PHA-activated porcine PBMCs or 293 cells producing high-titer PERV-A/C were cultured on hanging cell culture inserts [24]. In these studies, co-cultivation with mitogen-activated PBMCs from two pigs resulted in the integration of PERVs proviral DNA in 293 cells [24]. However, no infection of human PBMCs with PERV-A/C was observed. Notably, a PERV-A/C variant containing a higher number of transcription factor binding sites in the long terminal repeat (LTR) was able to infect human PBMCs [9,11].

In a separate study, co-culture experiments using a porous membrane setup—with 293 cells producing high-titer PERV-A/C or PERV-B in the upper chamber and mouse 3T6 cells in the lower chamber—did not result in infection of the mouse cells, as no PERV provirus was detected by a PCR specific to each PERV subtype [25].

However, this set-up does not take into account the cell-to-cell transmission of viruses, which is a common mode of retroviral infection (see Section 5).

## 5. Cell-to-Cell Transmission of Retroviruses

Retroviruses can infect target cells either by cell-free transmission or by direct cell-to-cell spread. Using cell-to-cell spread retroviruses may circumvent humoral immunity in vivo. The cell-to-cell spread of the human immunodeficiency virus type 1 (HIV-1) and the human T-cell lymphotropic virus type 1 (HTLV-1) are well studied (for reviews see [26,27]). Cell-to-cell spread takes place at specialized contact-induced virological synapses between infected and uninfected cells. In the case of HIV-1, the large extracellular loop of CD63 and the transmembrane envelope protein gp41 of HIV-1 are essential for the establishment of viral synapses [28]. Gamma-retroviruses are not well studied in this context. The *murine leukemia virus* (MuLV), a close relative to PERVs, utilizes virus-induced filopodia for efficient dissemination [29]. These filopodia originate from non-infected cells and interact, through their tips, with infected cells. The viral envelope glycoprotein (ENV) on an infected cell interacts with the receptor molecules on a target cell and generates a stable bridge. Viruses then move along the outer surface of the filopodial bridge toward the target cell. Virus spreading has been shown to be sensitive to reagents affecting the actin/myosin machinery [29]. The assembly of MuLV is directed towards sites of cell–cell contact [30]. There are two mechanisms: surface-based transmission and de novo assembly at the site of contact [31]. B cells infected with Friend MuLV (F-MuLV) have been shown to form virological synapses within the lymph node of living mice, as shown by intravital staining [32,33]. In vivo virological synapses showed one striking difference when compared with synapses in vitro: whereas in vitro synapsis formation of F-MuLV is entirely driven by ENV–receptor interactions, in vivo F-MuLV-infected primary B cells selectively interact with CD4^+^ T cells and less with CD8^+^ T cells, despite the fact that both cell types express similar mCAT-1 receptor levels and are equally susceptible to cell-free F-MuLV. Therefore, other factors in addition to the interaction of ENV and receptor must contribute to the selectivity of F-MuLV spreading in vivo [33]. To summarize, cell-to-cell spread is an important mode of retrovirus transmission. PERVs are likely to utilize cell-to-cell transmission to infect target cells; however, the underlying mechanism remains to be determined.

## 6. Use of Selection Marker-Resistant Human Cells

The third assay is based on the co-culture of pig cells with human cells that have been transfected with a selection marker (Figure 1C). This method is the easiest to handle and yields the purest population of human cells. These human cells are made resistant against a selection medium. An example of such a selection marker is the neomycin phosphotransferase (neo) gene, which confers resistance to geneticin or G418, a toxic aminoglycoside, in eukaryotic cells [34]. Another example is hygromycin B, an aminocyclitol antibiotic produced by *Streptomyces*. Hygromycin B phosphotransferase isolated from *Escherichia coli* has been shown to permit direct selection for hygromycin B resistance following the transfection of eukaryotic cell lines [34].

To study the potential for PERVs infection of human cells, we first applied this method in 2005 and later in 2008 [25,35]. Human 293 cells and mouse 3T6 cells were rendered neomycin-resistant (293neo^+^ and 3T6neo^+^, respectively) and co-cultured for five days with 293 cells, producing high-titer PERV-A/C. Following co-cultivation, a selection medium was applied to eliminate PERVs-producing 293 cells. Although no PERVs-specific sequences were detected in the murine 3T6 target cells, co-cultivation of 293neo^+^ cells with PERVs-producing 293 cells consistently resulted in infection [25].

In a subsequent 2008 study, 293neo^+^ cells were co-cultured with insulin-producing islet cells from German Landrace pigs. It was demonstrated that these porcine islet cells did not produce infectious, human-tropic PERVs [35]. PERVs infection was assessed using a PERVs-specific PCR, and successful elimination of porcine cells after selection was confirmed via a PCR targeting the pig mitochondrial COII gene.

Later, in 2021, Kono et al. [36] adapted this method by using hygromycin-resistant 293 cells to further investigate PERVs infectivity. After co-cultivation with PK15 cells, PERVs proviruses were found in hygromycin-resistant 293 cells by a PCR, and virus particles were found by electron microscopy. The absence of the porcine alpha-1,3-galactosyltranferase gene indicated that all PK15 cells were removed.

The ability to infect human cells varies among pig species due to differences in the number of infectious proviruses [16,37]. To summarize: in addition to its advantages over the other two methods—specifically, (i) avoiding treatments like gamma irradiation, which could impact PERVs expression and replication in donor cells, (ii) enabling direct contact between human and pig cells, (iii) allowing detection of both, free virus and cell-to-cell transmission, and (iv) allowing for the complete removal of pig cells, which was not fully achieved in the first assay—this method also offers the added benefits of not requiring expensive equipment and being less time-consuming.

## 7. Infectivity Assessment Using High-Throughput Sequencing Technologies

Kono et al. [36] determined porcine genome sequences and evaluated the infectivity of PERVs using high-throughput sequencing technologies. As not all integrated proviruses produce infectious human-tropic viruses due to some being defective, understanding the expression of full-length proviruses is crucial. However, the presence of full-length mRNA does not necessarily indicate that the provirus can produce infectious, human-tropic viral particles. The expression of spliced mRNA is essential for the translation of ENV proteins and the subsequent release of viral particles.

Furthermore, RNA sequencing was performed on both porcine cells (detecting all integrated proviruses) and PERVs-infected human cells (detecting sequences of proviruses able to produce infectious human-tropic virus), and reads mapped to PERVs sequences were examined. The normalized number of reads mapped to PERVs regions was able to predict the infectivity of PERVs, indicating that it would be useful for evaluating the PERVs infection risk prior to the transplantation of porcine products [36]. However, this approach is also limited by the selection of sequenced cells. Transformed human cell lines are permissive to PERVs infection, whereas primary human cells are typically not. Therefore, it remains unclear whether this method can reliably estimate infection potential prior to clinical application.

## 8. What Do the Assays Tell Us?

Regardless of the assay performed, including the high-throughput sequencing approach, the selection of donor pig cells and recipient human cells is crucial to the experimental outcome. The results remain inherently limited, and depend on the type of pig cells and human cells used. A cell line like PK15 releases infectious human-tropic PERVs, whereas normal pig lymphocytes do not [38]. However, after stimulation with a mitogen, pig PBMCs may release an infectious virus [7,39,40]. This depends on the pig breed; minipigs more often release an infectious human-tropic virus, usually PERV-A/C [41]. Other pig primary cells also release PERVs infectious for human cells [6], but PBMCs stimulated with a T cell mitogen and 12-O-tetradecanoyl-phorbol-13-acetate (TPA) were the best producers [7,38,39,40,41,42]. Concerning the target cells, human 293 cells are highly susceptible to PERVs infection, likely due to the loss of intracellular restriction factors [43]. In contrast, primary human cells have not been successfully infected with PERVs released from pig cells yet. Only a human cell-adapted PERV, which had a high replication rate due to an increased number of transcription factor binding sites in its long terminal repeats (LTRs) [11], was able to achieve infection of primary human PBMCs [4].

Transplanting a vascularized pig organ involves the transfer of various cell types, including stem cells. Stem cells are known for their high expression of endogenous retroviruses, which decreases as they differentiate [44]. The transplant recipient also consists of diverse cell types, and it remains uncertain whether certain primary human cells exhibit susceptibility comparable to 293 cells. Of particular interest is evaluating whether human stem cells can be infected by PERVs.

## 9. Future Development

Therefore, these assays have limited utility in assessing the risk posed by PERVs in transplants derived from specific pig breeds. Using alternative cell types as pig donors or human recipients is unlikely to substantially improve these assays, as not all donor–recipient combinations can be tested. Only the use of stem cells offers the potential for a significant increase in knowledge.

Similarly, combining in vivo and in vitro assays is not expected to enhance predictive accuracy. The limitations of in vivo models are well documented. Small animals cannot be infected due to their lack of the PERV receptor [45]. When transplanting pig organs into non-human primates, no transmission of the virus was observed despite strong immunosuppression and long-term survival. No PERV infections were observed when non-human primates were inoculated with high doses of PERVs. The main reason for this was the absence of fully functional PERV receptors in these models [46,47]. As a result, there is currently no definitive or reliable experimental method to assess the risk of PERVs transmission; only long-term monitoring of actual xenotransplant recipients can ultimately provide conclusive answers.

## 10. Conclusions

The assay based on the co-culture of pig cells with human cells that have been transfected with a selection marker best simulates the conditions of in vivo xenotransplantation (Table 1). This method is the easiest to handle and yields the purest population of human cells. However, like the other assays, it only indicates whether a specific pig cell type releases PERVs and whether a specific human cell type is susceptible to infection. A negative infection result does not necessarily reflect the in vivo situation in which a transplanted organ consists of multiple pig cell types interacting with a diverse range of human cells within a living organism. The capability of this assay as well as the other assays to assess the risk posed by PERVs in transplants derived from specific pig breeds is limited. Knowledge of these limitations is important not only for scientists in the field, but also for authorities regulating clinical applications of xenotransplantation.

## Figures and Tables

**Table 1:** Advantages and disadvantages of infection assays.

Assay	Advantages	Disadvantages
Co-cultivation with pig cells after gamma-irradiation	- Accounts for both cell-free and cell-to-cell transmission	- Requires gamma irradiation - Gamma irradiation may alter virus expression
Separation of pig and human cells by porous membranes	- No need to inactivate virus-producing cells - Easy removal of cell culture inserts	- Does not capture cell-to-cell transmission
Use of selection marker-resistant human cells	- Avoids the need for gamma irradiation, preserving natural PERVs expression and replication - Enables direct contact between pig and human cells - Allows detection of both free virus and cell-to-cell transmission - Enables complete removal of pig cells - Cost-effective and less time-consuming; does not require specialized equipment	- Requires target cells with a selection marker and selection medium

## Data Availability

No new experimental data were created.

## Abbreviations

The following abbreviations are used in this manuscript:

CRISPR/Cas	Clustered Regularly Interspaced Short Palindromic Repeats/CRISPR-associated
DPF	Designated pathogen-free
DSB	Double strand breaks
Env	Envelope
F-MuLV	Friend murine leukemia virus
HIV-1	Human immunodeficiency virus type 1
HTLV-1	Human T-cell lymphotropic virus type 1
NGS	Next-generation sequencing
PBMCs	Peripheral blood mononuclear cells
PERVs	Porcine endogenous retroviruses
PK15	Porcine kidney cells 15
ROS	Reactive oxygen species
TPA	12-O-tetradecanoyl-phorbol-13-acetate
